# Rapid spread of MPXV clade Ib with high genetic relatedness among men who have sex with men, Berlin, Germany, week 50 2025 up to week 10 2026

**DOI:** 10.2807/1560-7917.ES.2026.31.12.2600235

**Published:** 2026-03-26

**Authors:** Alexander Bartel, Klaus Jansen, Ronja Boberg, Julia Bitzegeio, Annika Brinkmann, Livia Schrick, Raskit Lachmann, Daniel Sagebiel, Andreas Nitsche, Claudia Ruscher, Janine Michel

**Affiliations:** 1 Surveillance and Epidemiology of Infectious Diseases Unit, State Office for Health and Social Affairs (SOHSA), Berlin, Germany; 2 Unit for HIV/AIDS, STI and Blood-borne Infections, Department of Infectious Disease Epidemiology, Robert Koch Institute, Berlin, Germany; 3 Center for Biological Threats and Special Pathogens 1, Highly Pathogenic Viruses, German Consultant Laboratory for Poxviruses, Robert Koch Institute, Berlin, Germany; 4 Unit for Gastrointestinal Infections, Zoonoses and Tropical Infections, Department of Infectious Disease Epidemiology, Robert Koch Institute, Berlin, Germany

**Keywords:** Mpox, MPXV, surveillance, outbreak, transmission, Clade Ib, Germany, MSM, men who have sex with men, sexual contact

## Abstract

Following the first detection of monkeypox virus (MPXV) clade Ib in Berlin, Germany, in December 2025, clade Ib rapidly predominated over clade IIb among notified mpox cases. The 35 clade Ib cases were primarily due to autochthonous transmission, with high genetic relatedness among strains circulating in men in Berlin, despite no identified epidemiological links. Sexual contact between men was reported as a potential source of infection in 28 cases, while for the remaining seven cases this information was unknown.

Since September 2023, the World Health Organization (WHO) has reported the rapid spread of the newly identified clade Ib of the monkeypox virus (MPXV), mainly in eastern Democratic Republic of Congo (DRC) and several other countries in East and Central Africa [1,2]. Unlike the previously described clade Ia, clade Ib has been associated with longer chains of human-to-human transmission, particularly through sexual contact [3]. Here, we describe the introduction and autochthonous spread of MPXV clade Ib in Berlin by comparing the epidemiological and phylogenetic characteristics of the first closely related clade Ib cases with clade IIb cases between week 45 2025 and week 10 2026.

## Autochthonous clade Ib transmission among MSM in Berlin

Between October 2024 and October 2025, 15 cases of mpox clade Ib were notified in Germany to the Robert Koch Institute (RKI, German national public health institute) [4,5]. Eleven cases were linked to travel to an African or Asian country. Four additional cases occurred as secondary household infections.

Since October 2025, autochthonous transmission of MPXV clade Ib has been reported in several European countries among men, acquired through sexual contact with other men [6,7]. From December 2025 (week 50/2025) until March 2026 (week 10/2026), 39 cases of mpox clade Ib were reported in Germany solely in men, mostly among men who have sex with men (MSM). Of the 39 clade Ib infections, 31 were probably acquired in Berlin; for seven cases this information is unknown.

**Figure 1:**
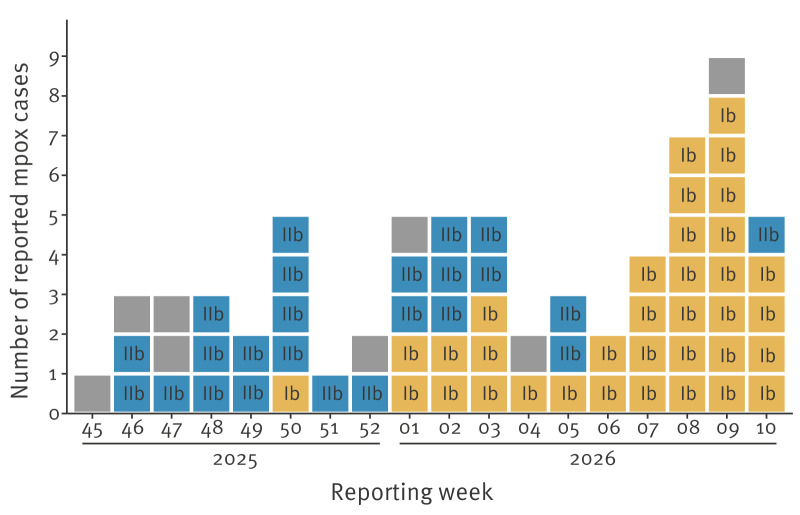
Epidemiological curve of all notified mpox cases by reporting week, Berlin, Germany, November 2025 (week 45)–March 2026 (week 10) (n = 67)

The first clade Ib case in Berlin, notified in week 50 2025, was linked to travel within Europe. This case was reported just before Berlin’s first locally acquired clade Ib case, notified in week 1 in 2026. However, based on epidemiological investigations, no link between the first autochthonous cases and the imported case could be established. Between weeks 1 and 5 in 2026, both clade Ib and clade IIb cases were reported in Berlin; notifications from week 6 onwards were predominantly identified as clade Ib (Figure 1). All 35 clade Ib cases were reported in men with a median age of 35 years (range: 21–52). Transmission of clade Ib in Berlin was primarily associated with sexual contact among men who self-identified as MSM (n = 28; 80%); for 20% (n = 7) this information was unknown. With only the initial case being import-associated, the clade Ib infections detected in Berlin are predominantly autochthonous (Table). Overall mpox case numbers in Berlin were substantially higher in 2026 than during the same period in previous years (weeks 1–10 during 2023: 10; 2024: 8; 2025: 27; 2026: 47).

Virus clades of reported mpox cases are labelled and indicated by colour with blue for clade IIb (n = 24), yellow for clade Ib (n = 35) and grey unknown (n = 8). The epidemiological curve was created using ggsurveillance in R [21].

**Table:** Characteristics and reported exposures of all notified mpox cases, Berlin, Germany, November 2025 (week 45)–March 2026 (week 10) (n = 67)

Characteristics	Overall n = 67		MPXV clade						
			Clade Ib n = 35		Clade IIb n = 24		Unknown n = 8		
	n	%	n	%	n	%	n		%
Age in years									
Median (IQR)	34.0 (28.0–40.0)		35.0 (28.0–40.0)		34.0 (30.5–40.0)		26.5 (24.0–32.0)		
Range	19–66		21–52		24–66		19–36		
Gender									
Female	0	0	0	0	0	0	0	0	
Male	67	100	35	100	24	100	8	100	
Diverse	0	0	0	0	0	0	0	0	
Hospitalisation									
Yes	5	7	2	6	1	4	2		25
No	60	90	32	91	22	92	6		75
Unknown	2	3	1	3	1	4	0		0
Vaccination^a^									
Vaccinated	31	46	19	54	10	42	2		25
One dose	9	NA	5	NA	2	NA	2		NA
Two doses	18	NA	12	NA	6	NA	0		NA
Doses unknown	4	NA	2	NA	2	NA	0		NA
Unvaccinated	29	43	11	31	13	54	5		63
Unknown	7	11	5	14	1	4	1		13
Place of infection^b^									
Berlin	55	82	27	77	20	83	8		100
Abroad	4	6	1	3	3	13	0		0
Unknown	8	12	7	20	1	4	0		0
Sexual contact									
Sex with men	58	87	28	80	22	92	8		100
Unknown	9	13	7	20	2	8	0		0

MPXV: monkeypox virus; NA: not applicable.

^a^ Any mpox vaccination with Modified Vaccinia Ankara-Bavarian Nordic (MVA-BN; also known as Jynneos or Imvanex) up to 2 weeks prior to infection.

^b^ Probable place of infection was determined through case ascertainment. If cases reported no travel, place of infection was counted as locally acquired (Berlin). Unknown place of infection was recorded if cases could not be reached. All cases reported to have their primary place of residence in Berlin.

## Assessment of clinical severity in clade Ib cases

To examine characteristics between clades, all mpox cases notified in Berlin from November 2025 until reporting week 10 in 2026 were compared (n = 67). For eight cases, clade information was unavailable. Cases infected with clade Ib or clade IIb were similar regarding age, sex, vaccination status, reported symptoms, mpox-related hospitalisation and probable infection route or exposure (Table). Overall, five of 65 cases were hospitalised, including two clade Ib cases and one clade IIb case.

Information from case ascertainment during routine surveillance and in-depth interviews from 28 of the 35 Berlin cases could not establish epidemiological links between the majority of cases. Transmission links could be ascertained for only six cases, resulting in three case pairs with household (one pair) or sexual contacts (two pairs). While eight cases reported mostly anonymous sexual contacts in 12 different sex-on-premises-venues (clubs, saunas and private parties), common sources of infection could not be identified.

## Genomic analysis of clade Ib transmission in Berlin

**Figure 2:**
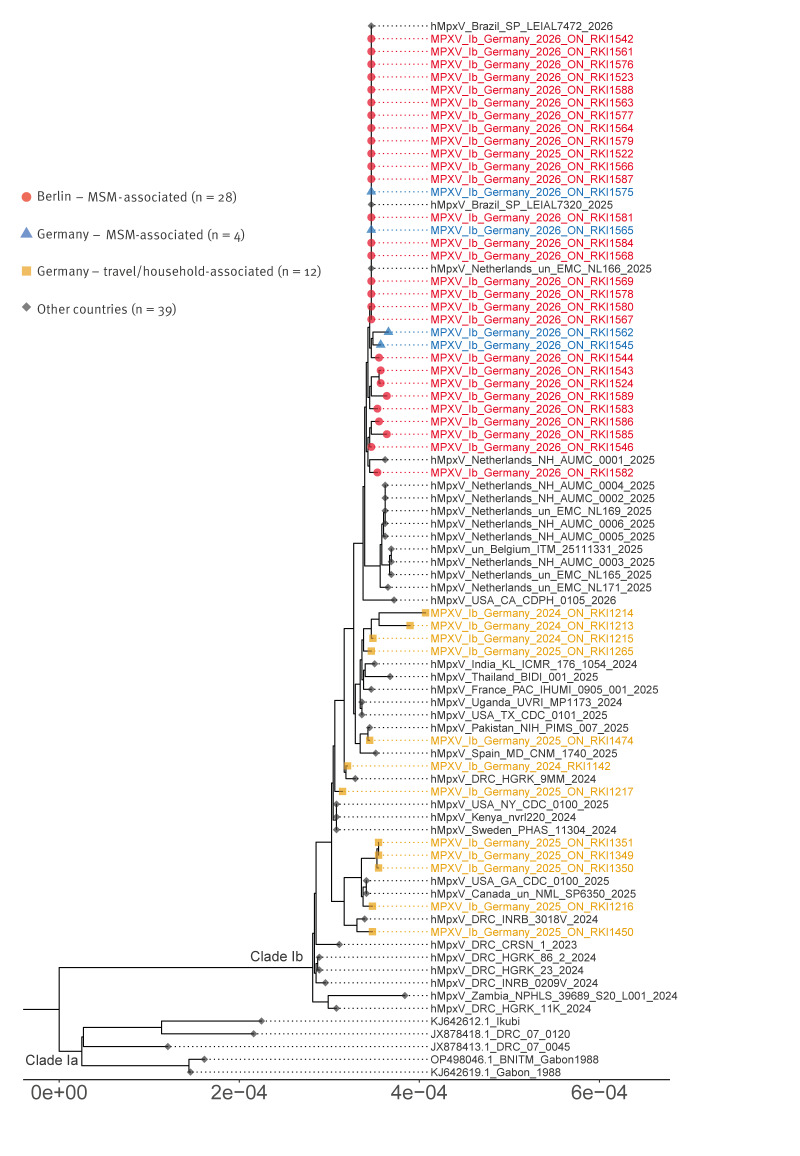
Phylogenetic tree of MPXV clade I sequences, Germany, October 2024–March 2026 (n = 44) compared with global sequences available on GISAID, November 2023–January 2026 (n = 39)

MPXV: monkeypox virus; MSM: men who have sex with men.

Although very few epidemiological links between cases could be identified, genomic sequencing provided evidence of a shared transmission network. Sequences were obtained from 28 of 35 strains from notified clade Ib cases in Berlin and all clade Ib cases (n = 4) reported in 2026 in other German federal states. These sequences from MSM-associated cases revealed a genetic cluster that differs from other German MPXV clade Ib sequences obtained from travel-associated cases (12/15 cases) and their household contacts from 2024 to 2025 (Figure 2). Sequences obtained from cases in Brazil, the Netherlands and Belgium from 2025 seem closely related to the Berlin cluster, suggesting an unknown epidemiological connection.

The tree is based on the MPXV core genome which spans 116,000 bp from K2R to A50R. The tree includes sequenced strains from MSM-associated mpox cases reported from Berlin (red circle), other German federal states (blue triangle), travel/household-associated mpox cases (yellow square) and other countries (grey, diamond). Non-German sequences were kindly provided by GISAID contributors, and are provided in the Supplement [22].

The observed transmission dynamics of clade Ib cases, due to their high genomic relatedness, differ substantially from the circulation of clade IIb strains. In 2025 in Berlin, various clade IIb lineages, e.g. E.1.1, F.2.1, F.4 and F.4.1, with high genetic diversity within lineages circulated concurrently over several months and thus formed multiple distinct persisting clusters in Berlin.

## Discussion

Berlin is a city that attracts MSM both nationally and internationally who seek sex on premises [8,9]. This was evident during the international mpox outbreak in 2022 (clade IIb), when Berlin represented a hotspot of epidemiological dynamics in Europe [10]. In February 2026, several countries informed the RKI via International Health Regulations (IHR) notifications and EpiPulse, the surveillance portal operated by the European Centre for Disease Prevention and Control (ECDC) [11], about mpox clade Ib cases in men believed to have been infected through sexual contacts in Berlin, including cases from Canada (n = 1), Czechia (n = 3), France (n = 1), Ireland (n = 1), Mexico (n = 1), Sweden (n = 1), Switzerland (n = 1) and the United Kingdom (n = 1).

Between 2022 and early 2025, several waves of mpox cases have previously been observed in German metropolitan areas [12]. At present, it is difficult to estimate whether the observed increase in case numbers will continue and how the distribution of clades could develop. Additionally, previous estimates of mpox case numbers assumed a substantial number of undiagnosed and unreported MPXV clade IIb infections [13,14]. However, their impact on the distribution of the different clades remains uncertain.

While the drivers of the currently observed predominance of clade Ib over clade IIb are still unknown, they may indicate a temporary infection or transmission advantage of clade Ib in a population with no prior exposure to clade Ib. In addition, prior mpox infection or vaccination within populations at risk are likely to influence the infection dynamics of clade Ib as well; unfortunately, information about prior infection was not available for our cases. Reinfection presenting with mild or inapparent clinical symptoms in individuals with a high number of (anonymous) sexual partners may additionally drive transmission in populations at risk [14,15].

Vaccination is the core preventive measure against mpox [16]. Gubela et al. assumed that immunity acquired during the mpox outbreak in 2022, as well as the accompanying vaccination campaign, may have created a temporary herd immunity among the population at risk for mpox infection, which may fade over time because of demographic changes and waning immunity [14]. Additionally, the increase in mpox cases in 2025 and 2026 in Berlin may indicate vaccination gaps following the clade IIb outbreak in 2022. Since 2023, 348 cases had information available about vaccination status. The proportion of cases who had received at least one dose of mpox vaccine before infection declined from 66% (50/76) in 2023 and 64% (39/61) in 2024 to 51% (86/170) in 2025 and 49% (20/41) in 2026. 

The Modified Vaccinia Ankara-Bavarian Nordic (MVA-BN; Imvanex) is a highly effective and well-tolerated vaccine against mpox that is available across all countries in the European Union (EU) [17]. The Standing Committee on Vaccination (STIKO) at the RKI recommends its use regardless of gender as an indication vaccination for people with an increased risk of infection (e.g. cis-men, trans and non-binary people who have sex with men and frequently change partners, and sex workers) as well as for post-exposure vaccination [18]. Particularly in light of the emergence of clade Ib in sexual networks, but also to protect against clade IIb, all individuals at elevated risk of MPXV infection should be offered vaccination in accordance with the STIKO recommendation in a timely manner [19,20]. This is particularly relevant in Berlin, where sex-positive venues are not always strictly oriented towards specific genders or sexual preferences, and where there is a great deal of fluidity and openness between these groups.

## Conclusion

Targeted vaccination efforts, accessible testing and continued genomic surveillance are essential to limit further spread, monitor the epidemiological situation of MPXV clade Ib in Germany and Europe, and inform appropriate public health measures.

## Data Availability

All data generated or analysed during this study are included in this published article. Case level data will not be shared due to privacy reasons. Mpox sequences are partially available and remaining sequences will be made available in the GISAID and GenBank databases by the Centre for Biological Threats, Highly Pathogenic Viruses, Robert Koch Institute.

## Supplementary Data

40000 Supplement
