# Supplementary material for: Rapid spread of MPXV clade Ib with high genetic relatedness among men who have sex with men, Berlin, Germany, week 50 2025 up to week 10 2026
Source: Euro Surveill. 2026 Mar 26;31(12):2600235. doi: 10.2807/1560-7917.ES.2026.31.12.2600235 (PMC13074487; doi:10.2807/1560-7917.ES.2026.31.12.2600235)
Supplement: Supplement [file 26-00235_BARTEL_Supplement.pdf]

Supplementary Appendix

"This supplementary material is hosted by Eurosurveillance as supporting information alongside the article [Rapid spread of MPXV clade Ib with high genetic related-ness among MSM, Berlin, Germany, week 50 2025 up to week 10 2026], on behalf of the authors, who remain responsible for the accuracy and appropriateness of the content. The same standards for ethics, copyright, attributions and permissions as for the article apply. Supplements are not edited by Eurosurveillance and the journal is not responsible for the maintenance of any links or email addresses provided therein."

All genome sequences and associated metadata supporting the findings of this study can be accessed through the persistent digital object identifier https://doi.org/10.55876/gis8.260316gx

In addition to the minted DOI, GISAID also communicates the aggregation of GISAID accession numbers (EPI_ISL_IDs) through the corresponding EPI_SET_260316gx identifier to facilitate both, the acknowledgment of all data contributors and the direct retrieval of the underlying data from GISAID used in this study.

MPox Virus Data Summary

GISAID Identifier	Digital Object Identifier	Number of individual viruses	Data Collection range	Number of countries/territories
EPI_SET_260316gx	https://doi.org/10.55876/gis8.260316gx	33	2023-11-02 to 2026-01-14	14
